# MRI-based clinical-radiomics-habitat model for predicting prognosis of hepatocellular carcinoma patients treated with HAIC

**DOI:** 10.3389/fonc.2026.1764150

**Published:** 2026-01-28

**Authors:** Jiaojiao Cao, Tianyi Zhu, Qichen Sun, Yiting Liu, Haiyang Yu, Xiaoxia Guo, Qin Liu, Xiaoyi Ding, Jian Wang, Zhiyuan Wu, Yu Zhou

**Affiliations:** 1 Department of Interventional Radiology, Ruijin Hospital, Shanghai Jiao Tong University School of Medicine, Shanghai, China; 2 Faculty of Medical Imaging Technology, College of Health Science and Technology, Shanghai Jiao Tong University School of Medicine, Shanghai, China; 3 Liver Center, Ruijin Hospital, Shanghai Jiao Tong University School of Medicine, Shanghai, China; 4 Department of Neurosurgery, Ruijin Hospital, Shanghai Jiao Tong University School of Medicine, Shanghai, China; 5 Department of Interventional Radiology, Affiliated Changshu Hospital of Nantong University, Suzhou, China

**Keywords:** habitat, HAIC, HCC, intratumoral heterogeneity, radiomics

## Abstract

**Background:**

Hepatocellular carcinoma (HCC) is a highly heterogeneous malignant tumor with generally poor prognosis. Hepatic arterial infusion chemotherapy (HAIC) serves as a crucial treatment modality for intermediate to advanced HCC, but its efficacy is significantly influenced by tumor heterogeneity and individual variability. This study aimed to develop an MRI-based Clinical-Radiomics-Habitat model for non-invasive prediction of early response to HAIC treatment.

**Methods:**

105 HCC patients who received HAIC treatment across two institutions were retrospectively analyzed. Tumor subregions were segmented on four preoperative MRI sequences, including T1-weighted imaging (T1WI) and contrast-enhanced T1WI (arterial late phase, portal venous phase, and delayed phase), from which image features were extracted. Clinical data, habitat analysis features, and radiomics features were collected to construct three distinct predictive models. Each model was internally validated using the bootstrap method, evaluated using multiple performance metrics, and employed to explore prognostic information.

**Results:**

Among the 105 patients, treatment responses included complete response (n=9), partial response (n=48), stable disease (n=34), and progressive disease (n=14), yielding responder and non-responder rates of 54.3% and 45.7%, respectively. With an AUC of 0.771 (95% CI 0.682–0.860), the Clinical–Radiomics–Habitat model performed better than both the Clinical model (0.633) and the Clinical–Radiomics model (0.747). Habitat imaging exhibits significant potential in analyzing tumor heterogeneity and predicting treatment early response in HCC patients.

**Conclusion:**

We established a multiparametric MRI-based Clinical-Radiomics-Habitat model for preoperative early response prediction in HAIC-treated HCC patients. This model may assist clinicians in optimizing personalized treatment decisions.

## 1 Introduction

Hepatocellular carcinoma (HCC) is a highly lethal but common malignancy, and its incidence continues to rise globally (1, 2). HCC not only reduces the patients’ life expectancy and life quality, but also places a heavy financial burden on them owing to the high cost of treatment. Most patients with HCC are not candidates for curative resection at the time of diagnosis because the tumor often presents asymptomatically (3); consequently, therapy options including radiotherapy, chemotherapy, and molecular targeted therapy should be considered more comprehensively for the management of such patients (4).

By direct administration of chemotherapeutic agents into the hepatic artery at high concentrations, hepatic arterial infusion chemotherapy (HAIC) is considered effective in tumor reduction, vascular thrombus control, and improvement in patient survival with a high treatment response rate (5), and has become an important therapy for HCC at an intermediate-to-advanced stage (6, 7). However, as responses to HAICs vary among individual patients due to tumor heterogeneity and tumor treatment-related factors, identifying optimal treatment strategies and using objective criteria for estimating the treatment response are considered key clinical challenges.

In recent years, habitat imaging analysis has gained attention as a novel tumor-assessment method. By partitioning tumors into biologically meaningful subregions, this technique quantifies intratumoral composition and spatial interactions and has been applied across various malignancies (8, 9). Wu et al. (10) demonstrated that a Computed Tomography (CT) -based habitat radiomics model outperformed the model based solely on clinical variables. However, few studies have explored habitat analysis for predicting outcomes following HAIC in HCC. Moreover, Magnetic Resonance Imaging (MRI) provides superior sensitivity for HCC detection and characterization compared with CT, avoids ionizing radiation, and is widely used in pretreatment evaluation (2, 11). Therefore, this study aimed to extract features from whole tumors and their internal habitats on MRI to predict HAIC treatment response in patients with HCC.

## 2 Materials and methods

### 2.1 Patients

Our study received approval from the Ethics Committee of Ruijin Hospital, Shanghai Jiao Tong University School of Medicine. We retrospectively reviewed all HCC patients who received HAIC from January 2021 to December 2024 at Ruijin Hospital and Ruijin Hospital North.

Patients were included if (1) HCC diagnosed according to the American Association for the Study of Liver Diseases (AASLD) guidelines; (2) age ≥18 years; (3) at least one measurable unresectable lesion; (4) ECOG performance status of 0–1; (5) Child–Pugh class A or B; (6) available contrast-enhanced MRI within 1 month before HAIC and within 1 month after the final treatment cycle. Exclusion criteria included (1) receipt of additional treatments during follow-up; (2) severe cardiac, pulmonary, or renal insufficiency; (3) history of other malignancies; (4) poor-quality imaging or incomplete clinical data.

**Figure 1:**
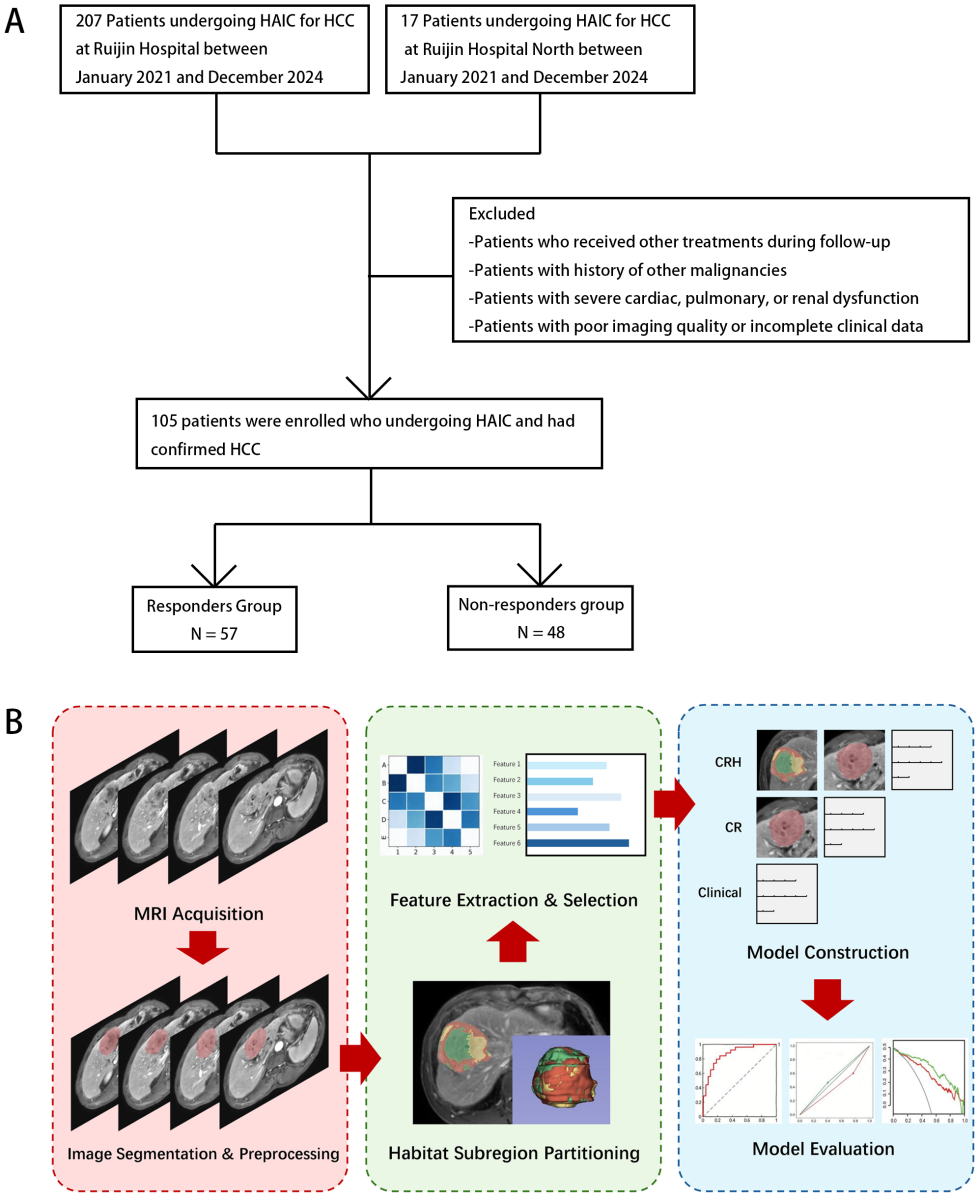
**(A)** Flowchart of patient enrollment and selection. **(B)** Workflow for predicting early response to HAIC in HCC.

We included 105 patients who met the eligibility criteria. A flowchart of our patient selection process is provided in **Figure 1A**.

### 2.2 HAIC therapy procedure

All patients underwent HAIC using a standardized FOLFOX regimen, repeated every 3 weeks. Under local anesthesia, femoral arterial access was obtained, and a conventional catheter with a microcatheter was selectively advanced into the tumor-feeding hepatic artery. The FOLFOX regimen consisted of oxaliplatin (85 mg/m²) administered via arterial infusion on day 1, followed by leucovorin (400 mg/m²) and fluorouracil (5-FU, 400 mg/m²) delivered intra-arterially, and continuous arterial infusion of 5-FU (2400 mg/m²) over 46 hours (days 2–3). No embolic agents were used.

### 2.3 Follow-up and response evaluation

Follow-up was conducted until the last recorded date (July 18, 2025). Treatment response was evaluated according to the modified Response Evaluation Criteria in Solid Tumors (mRECIST) criteria (12) based on MRI obtained within 1 month after completion of HAIC. Responses were categorized as complete response (CR), partial response (PR), stable disease (SD), or progressive disease (PD). Patients were subsequently grouped as having objective response (OR = CR + PR) or no objective response (nOR = SD + PD). Two radiologists specializing in abdominal interventional radiology (8 and 3 years of experience) independently evaluated all cases while blinded to clinical data; disagreements were resolved through consensus with a highly experienced radiologist (25 years of experience).

### 2.4 MRI acquisition

The workflow of our study is showed in **Figure 1B**. Four T1-weighted imaging (T1WI) sequences were included: pre-contrast (PRE), arterial late phase (LAP), portal venous phase (PVP), and delayed phase (DP). MRI scanners consisted of the uMR 790 3.0T, Philips Ingenia 3.0T, and GE SIGNA Architect AIR 3.0T. Detailed imaging parameters are provided in the **Supplementary Material S1**. Raw DICOM files were obtained from the Picture Archiving and Communication System (PACS) system for analysis.

### 2.5 Image segmentation and preprocessing

PVP images obtained prior to HAIC were imported into a software named 3D Slicer (version 5.2.2; www.slicer.org), and tumors were manually segmented by two radiologists (3 and 7 years of experience). Regions of Interest (ROIs) were delineated slice-by-slice.

To minimize variability across scanners and sequences, all images underwent N4 bias-field correction and were resampled to 1×1×1 mm. Registration was then conducted using ANTs (https://antsx.github.io/ANTs/), followed by min–max intensity normalization. All image processing was performed using Python (version 3.8.16; https://www.python.org). Following inter-sequence registration, all MRI sequences were spatially aligned; therefore, the tumor ROIs delineated on the portal venous phase were automatically mapped and correspondingly synchronized to the same anatomical locations on the remaining sequences.

### 2.6 Habitat subregion partitioning

**Figure 2:**
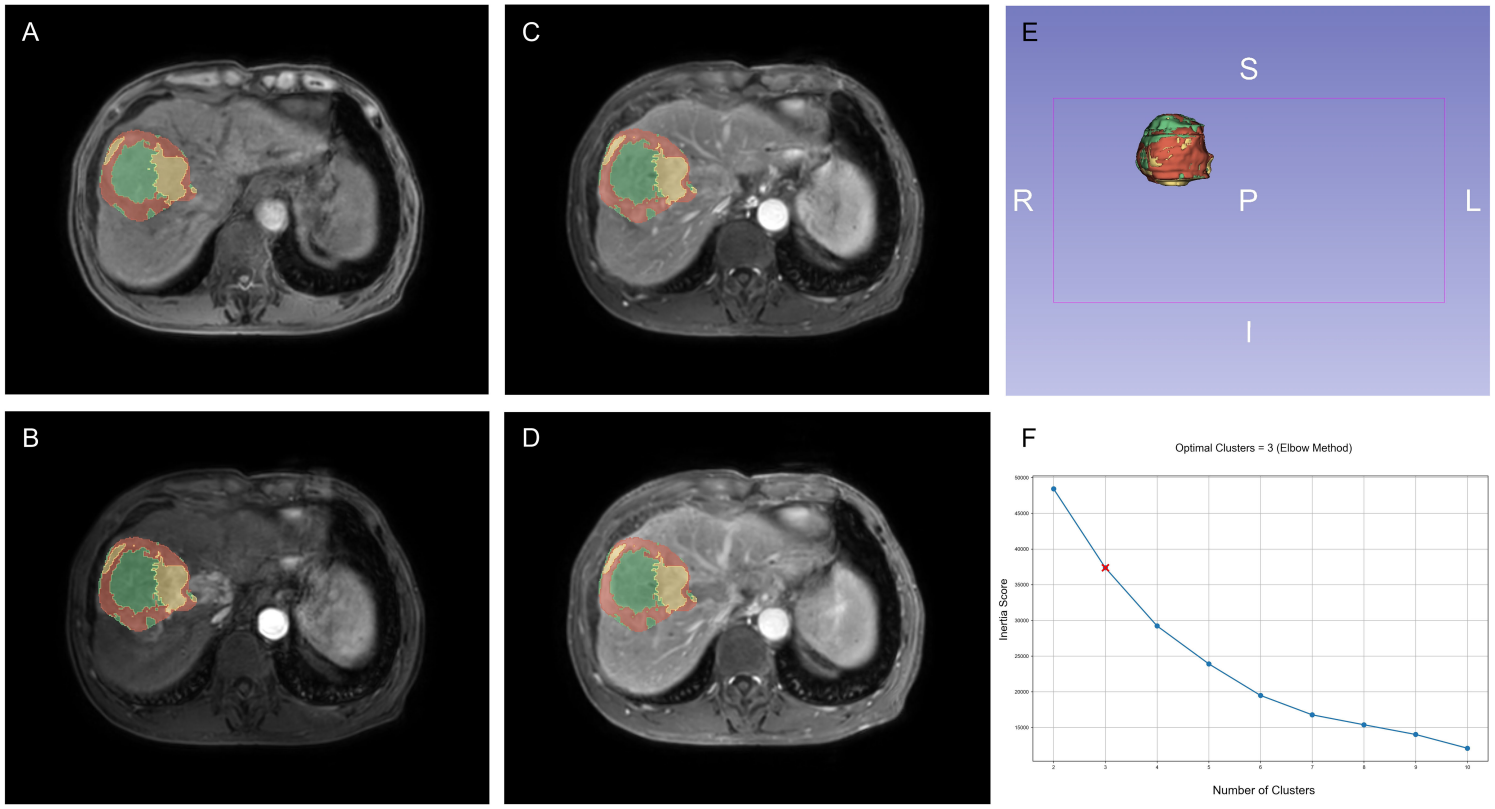
Habitat subregion segmentation across multiphase MRI and clustering validation. **(A)** Pre-contrast. **(B)** Arterial phase. **(C)** Portal venous phase. **(D)** Delayed phase. **(E)** 3D view of the ROI divided into three habitat subregions. **(F)** Determination of the optimal number of clusters using the Elbow method.

Habitat subregion partitioning was performed using the open-source Python-based HABIT toolkit (https://github.com/lichao312214129/HABIT.git). For each tumor, unsupervised K-means clustering was applied to the voxel-wise intensity features within the ROI. The optimal number of clusters was then determined using the Elbow method by computing a value called the inertia that represents the sum of squared distances from each sample to its nearest cluster center. The inertia was calculated for k-means with k ranging from 2 to 10; we identified k as the point where the reduction of inertia became steady in the inertia–k curve. In our analyses, we selected k = 3. (**Figure 2F**). The intratumoral heterogeneity of HCC, as characterized by distinct habitat subregions, is shown in **Figures 2A–E**.

### 2.7 Feature extraction

Three types of features were considered: clinical features, radiomic features, and habitat features. Clinical features included demographic information, laboratory test data, and imaging features of tumors and were screened by univariate logistic regression analyses followed by multivariable logistic regression analyses. Radiomic features were extracted from whole-tumor ROIs whereas habitat features were extracted from tumor subregions, using PyRadiomics (https://pypi.org/project/pyradiomics/).

The stability of the extracted features was tested in 30 randomly selected patients by performing three repetitive extractions to calculate its coefficient of variation (CV), which was less than 15%. Features showing CV < 15% were considered stable. To further assess the inter-observer reproducibility of feature extraction, intraclass correlation coefficient (ICC) was used, and radiomic features with ICC < 0.8 were excluded. After normalization process was completed, highly correlated features (|r| > 0.9) were considered redundant and removed. Feature selection was done using recursive feature elimination (RFE) process followed by LASSO regression analysis (13) with 10-fold cross-validation technique after which feature selection was done based on iterative model building approach.

### 2.8 Model construction

The three types of features were incorporated sequentially to build three models: a clinical model, a clinical-radiomics (CR) model, and a clinical-radiomics-habitat (CRH) model with corresponding risk scores. We internally validated the models using 1,000 times bootstrap resampling. Given the small sample size in our study, bootstrap resampling was implemented to make sure that the evaluation of the models was robust (14, 15).

To assure feature independence and complementary information, we performed Spearman correlation and variance inflation factor (VIF) analyses with the combined features of the CR and CRH models. Severe redundancy indicated by |r|>0.8, and significant multicollinearity was indicated by VIF >10. Here, |r| is the absolute value of the Spearman correlation coefficient measuring the linear association between variables and VIF reflects how much a parameter estimate is increased due to multicollinearity.

### 2.9 Statistical analysis

Python (version 3.10.19; https://www.python.org) was used for all statistical analyses, with key functions from the scikit-learn, lifelines, matplotlib, and scipy libraries. For continuous variables, we reported those with a normal distribution as mean ± standard deviation (SD), while non-normally distributed ones were summarized as median (interquartile range). Categorical variables were presented as counts and percentages. For inter-group comparisons, independent t-test or Mann–Whitney U test according to distributions was used for continuous variables, and chi-squared test was used for categorical variables.

We assessed model discrimination with receiver operating characteristic (ROC) curves, area under the curve (AUC), and confusion matrices. Calibration and reliability were checked using calibration plots, and decision curve analysis was used to explore clinical utility. When comparing AUCs across models, we applied DeLong’s test. A two-sided p-value < 0.05 was considered statistically significant.

## 3 Results

### 3.1 Patient characteristics

Of 218 HCC patients who received HAIC, 47 were excluded due to concomitant malignancies, 21 for poor image quality or missing clinical data, and 45 for incomplete follow-up (**Figure 1**). Ultimately, 105 patients were included (mean age 57.3 ± 11.7 years; 97 male [92.4%], 8 female [7.6%]). According to treatment response, 9 achieved CR, 48 PR, 34 SD, and 14 PD. Overall, 57 patients (54.3%) were classified as OR, and 48 (45.7%) as nOR. Baseline characteristics are summarized in **Table 1**. Compared with the nOR group, the OR group showed notable differences in vascular invasion and tumor diameter; other baseline variables were similar (**Table 2**). Here, vascular invasion was defined as macrovascular invasion, specifically referring to the involvement of the portal vein and hepatic vein.

**Table 1:** Patients’ characteristics.

Variable	(Total) N=105
Age(years)	
<60	63(60.00)
≥60	42(40.00)
Sex	
Male Female	97(92.38) 8(7.62)
HBV Infection	
Negative	6(5.71)
Positive	99(94.29)
ECOG PS	
0	105(100.00)
1	0(0.00)
Child-Pugh class	
A	63(60.00)
B	42(40.00)
Ascites	
No	47(44.76)
Yes	58(55.24)
PT(s)	
≤13	78(74.29)
>13	27(25.71)
BCLC stage	
A	4(3.81)
B	14(13.33)
C	87(82.86)
ALBI grade	
1	18(17.14)
2	83(79.05)
3	4(3.81)
NLR	
<3	64(60.95)
≥3	41(39.05)
Vascular Invasion	
No	37(35.24)
Yes	68(64.76)
Extrahepatic Metastasis	
No	57(54.29)
Yes	48(45.71)
Tumor Number	
Single	42(40.00)
Multiple	63(60.00)
d_tumor(cm)	
≤10	51(48.57)
>10	54(51.43)
CRAFITY Score	
0	4(3.81)
1	46(43.81)
2	55(52.38)
AFP(ng/mL)	
≤20	23(21.90)
20-200	21(20.00)
≥200	61(58.10)
CRP(mg/L) [median (IQR)]	11[3.6,29.3]
PIVKA-II(mAU/mL)	
≤40	8(7.62)
(40, 300]	8(7.62)
>300	89(84.76)
BMI(kg/m^2^) [median (IQR)]	22.65[21.06,24.765]
Number of HAIC Sessions [median (IQR)]	3[2,4]
Response	
CR+PR	57(54.29)
SD+PD	48(45.71)

Unless otherwise indicated, the data are numbers of patients, with percentage in parentheses. For continuous variables, data are expressed as median (interquartile range, IQR).

HBV, hepatitis B virus; Child-Pugh, Child-Turcotte-Pugh classification; ALBI, Albumin-Bilirubin grade; NLR, Neutrophil-to-Lymphocyte Ratio; AFP, alpha-fetoprotein; PIVKA-II, Protein Induced by Vitamin K Absence or Antagonist-II; ECOG PS, Eastern Cooperative Oncology Group Performance Status; CRP, C-reactive protein; BMI, Body Mass Index; CRAFITY Score, C-reactive protein and alpha-fetoprotein in immunotherapy; CR, complete response; PR, partial response; SD, stable disease; PD, progressive disease; HAIC, Hepatic Arterial Infusion Chemotherapy.

**Table 2:** Baseline characteristics of effective and ineffective groups in hepatocellular carcinoma patients treated with HAIC.

Variable	OR (CR+PR, n=57)	nOR (PD+SD, n=48)	P value
HBV Infection			0.2887
Negative	2 (3.51)	4 (8.33)	
Positive	55 (96.49)	44 (91.67)	
Child-Pugh class			0.749
A	35 (61.40)	28 (58.33)	
B	22 (38.60)	20 (41.67)	
Ascites			0.3274
No	28 (49.12)	19 (39.58)	
Yes	29 (50.88)	29 (60.42)	
PT (s)			0.7683
≤13	43 (75.44)	35 (72.92)	
>13	14 (24.56)	13 (27.08)	
BCLC stage			0.4111
A	2 (3.51)	2 (4.08)	
B	6 (10.53)	8 (16.33)	
C	49 (85.96)	38 (79.59)	
ALBI grade			0.6501
1	10 (17.54)	8 (16.67)	
2	46 (80.70)	37 (77.08)	
3	1 (1.75)	3 (6.25)	
NLR			0.3647
<3	37 (64.91)	27 (56.25)	
≥3	20 (35.09)	21 (43.75)	
Vascular Invasion			0.037
No	15 (26.32)	22 (45.83)	
Yes	42 (73.68)	26 (54.17)	
Extrahepatic Metastasis			0.6776
No	32 (56.14)	25 (52.08)	
Yes	25 ( (43.86)	23 (47.92)	
Tumor Number			0.6313
Single	24 (42.11)	18 (37.50)	
Multiple	33 (57.89)	30 (62.50)	
d_tumor (cm)			0.0024
≤10	36 (63.16)	15 (31.25)	
>10	21 (36.84)	33 (68.75)	
CRAFITY Score			0.9264
0	3 (5.26)	1 (2.08)	
1	24 (42.11)	22 (45.83)	
2	30 (52.63)	25 (52.08)	
AFP (ng/mL)			0.1495
≤20	14 (24.56)	9 (18.75)	
20-200	14 (24.56)	7 (14.58)	
≥200	29 (50.88)	32 (66.67)	
PIVKA-II (mAU/mL)			0.7311
≤40	4 (7.02)	4 (8.33)	
(40, 300]	4 (7.02)	4 (8.33)	
>300	49 (85.96)	40 (83.33)	
Number of HAIC Sessions [median (IQR)]	3[2,4]	3[2,3]	0.5445

Unless otherwise indicated, data are presented as the number of patients [percentage in parentheses]; continuous variables are expressed as median (interquartile range, IQR).

OR (CR+PR): “OR” stands for “ Objective Response,” representing the treatment-responsive group; nOR (PD+SD): “nOR” stands for “non-Objective Response,” representing the treatment-nonresponsive group.

HBV, hepatitis B virus; Child-Pugh, Child-Turcotte-Pugh classification (i.e., Child-Pugh classification for liver function); ALBI, Albumin-Bilirubin grade; NLR, Neutrophil-to-Lymphocyte Ratio; AFP, alpha-fetoprotein; PIVKA-II, Protein Induced by Vitamin K Absence or Antagonist-II; CRAFITY Score, C-reactive protein and alpha-fetoprotein score in immunotherapy; HAIC, Hepatic Arterial Infusion Chemotherapy; CR, complete response; PR, partial response; SD, stable disease; PD, progressive disease; CI, Confidence Interval.

### 3.2 Radiomics and habitat feature extraction

**Figure 3:**
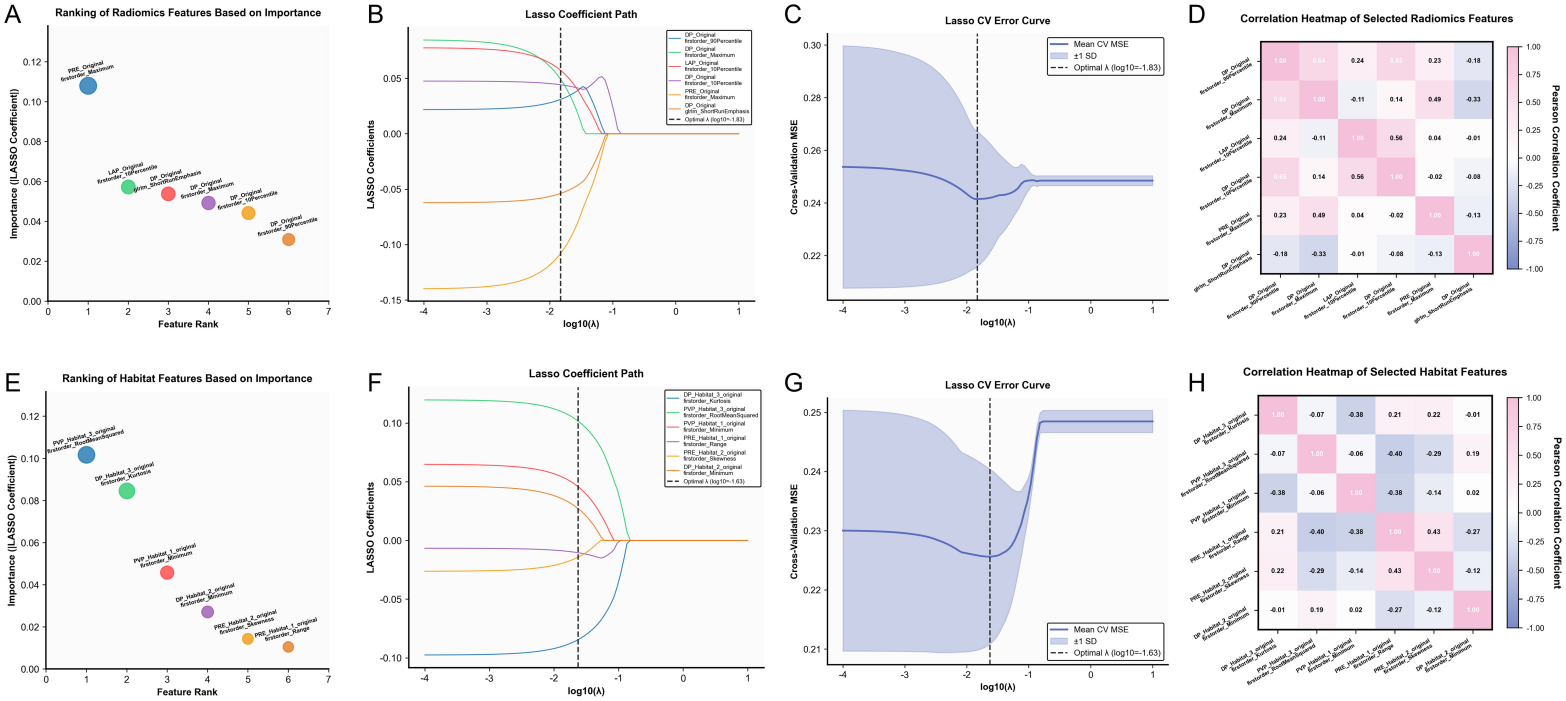
Feature selection of radiomics and habitat features. **(A, E)** Bubble plots showing feature importance for radiomics **(A)** and habitat **(E)** features. **(B, F)** LASSO coefficient paths for radiomics **(B)** and habitat **(F)** features. **(C, G)** Cross-validation error curves for radiomics **(C)** and habitat **(G)** feature selection. **(D, H)** Spearman correlation heatmaps for radiomics **(D)** and habitat **(H)** features.

A total of 428 radiomics features and 1,140 habitat features were initially extracted per patient. For each sequence, 93 features were extracted from both the whole-tumor ROI and each habitat subregion, including 18 first-order features, 24 GLCM features, 14 GLDM features, 16 GLRLM features, 16 GLSZM features, and 5 NGTDM features. Additionally, 14 shape features were calculated for each ROI, and habitat volume and proportion were recorded. Details of the feature extraction process are provided in **Supplementary Material S2**. After feature selection, six radiomics features (DP_Original_firstorder_10Percentile, DP_Original_glrlm_ShortRunEmphasis, DP_Original_firstorder_90Percentile, DP_Original_firstorder_Maximum, LAP_Original_firstorder_10Percentile, PRE_Original_firstorder_Maximum) and six habitat features (DP_Habitat3_firstorder_Kurtosis, PVP_Habitat3_firstorder_RootMeanSquared, PVP_Habitat1_firstorder_Minimum, PRE_Habitat1_firstorder_Range, PRE_Habitat2_firstorder_Skewness, DP_Habitat2_firstorder_Minimum) were retained as the most predictive (**Figure 3**).

### 3.3 Construction of predictive models

Univariate and multivariate logistic regression identified Vascular Invasion (p=0.015) and d_tumor (p=0.037) as independent clinical predictors (**Table 3**). These two factors were used to construct the clinical model, while the CR model was built by combining clinical features with the selected radiomics features. The CRH model was further developed by incorporating habitat analysis features. Risk scores were calculated using binary logistic regression as follows:

**Table 3:** Univariate and multivariate logistic regression analyses of the risk factors for treatment response to HAIC in patients with hepatocellular carcinoma (HCC).

Variable	Univariable analysis		Multivariable analysis	
	OR (95%CI)	P-value	OR (95%CI)	P-value
Age(years)				
<60	Reference			
≥60	1.212 (0.552 – 2.660)	0.631		
Sex				
Male	Reference			
Female	2.706 (0.520 – 14.078)	0.237		
HBV Infection				
Positive	Reference			
Negative	0.400 (0.070 – 2.286)	0.303		
Child-Pugh class				
A	Reference			
B	0.880 (0.402 – 1.926)	0.749		
Ascites				
Yes	Reference			
No	1.474 (0.677 – 3.206)	0.328		
PT(s)				
≤13	Reference			
>13	0.877 (0.365 – 2.107)	0.768		
BCLC stage				
A	1.333 (0.144 – 12.369)	0.800		
B	Reference			
C	1.719 (0.550 – 5.376)	0.352		
ALBI grade				
1	Reference			
2	0.995 (0.357 – 2.773)	0.992		
3	0.267 (0.023 – 3.080)	0.290		
NLR				
<3	Reference			
≥3	0.695 (0.316 – 1.528)	0.366		
Vascular Invasion				
No	0.422 (0.186 – 0.957)	0.039	0.324 (0.131 – 0.801)	0.015
Yes	Reference		Reference	
Extrahepatic Metastasis				
No	1.178 (0.545 – 2.546)	0.678		
Yes	Reference			
Tumor Number				
Single	1.212 (0.552 – 2.660)	0.631		
Multiple	Reference			
d_tumor(cm)				0.037
≤10	Reference		Reference	
>10	0.265 (0.118 – 0.598)	0.001	0.220 (0.092 – 0.526)	< 0.001
CRAFITY Score				
0	2.500 (0.245 – 25.556)	0.440		
1	0.909 (0.415 – 1.993)	0.812		
2	Reference			
AFP(ng/mL)				
≤20	Reference			
20-200	1.286 (0.374 – 4.419)	0.690		
≥200	0.583 (0.219 – 1.547)	0.278		
CRP(mg/L)	1.002(0.991-1.014)	0.684		
PIVKA-II((mAU/mL)				
≤40	0.816 (0.192 – 3.471)	0.783		
(40, 300]	0.816 (0.192 – 3.471)	0.783		
>300	Reference			
BMI(kg/m2)	0.982 (0.868 – 1.112)	0.779		
Number of HAIC Sessions	1.214 (0.903 – 1.632)	0.199		

HCC, Hepatocellular carcinoma; HBV, Hepatitis B virus; Child-Pugh, Child-Turcotte-Pugh classification; ALBI, Albumin-Bilirubin grade; NLR, Neutrophil-to-lymphocyte ratio; AFP, Alpha-fetoprotein; PIVKA-II, Protein induced by vitamin K absence or antagonist-II; CRAFITY Score, C-reactive protein and alpha-fetoprotein combined score in immunotherapy; HAIC, Hepatic arterial infusion chemotherapy; OR, Odds ratio; CI, Confidence interval.

*P < 0.05 was considered statistically significant.

Reference group explanation: The reference category for each categorical variable is indicated in the table (e.g., “Male” as the reference group for Sex, “Positive” as the reference group for HBV Infection), and the OR value represents the relative risk of treatment response in the respective group compared to the reference group.


$$\begin{array}{c} Clinical\text{ Model score}=\;0.6212\;+\;0.4103\;\\ \times \text{ Vascular Invasion }-\;0.0652\;\times \text{ d}\_\text{tumor} \end{array}$$

$$\begin{array}{c} CR\text{ Model score}\\ =0.7242\;+\;0.3415\;\times \text{ Vascular Invasion }-\;0.0694\;\times \text{ d}\_\text{tumor }\\ +\;0.1967\;\times \text{ DP}\_\text{Original}\_\text{original}\_\text{firstorder}\_10\text{Percentile }\\ -\;0.2450\;\times \text{ DP}\_\text{Original}\_\text{original}\_\text{glrlm}\_\text{ShortRunEmphasis }\\ +\;0.1234\;\times \text{ DP}\_\text{Original}\_\text{original}\_\text{firstorder}\_90\text{Percentile }\\ +\;0.2326\;\times \text{ DP}\_\text{Original}\_\text{original}\_\text{firstorder}\_\text{Maximum }\\ +\;0.1769\;\times \text{ LAP}\_\text{Original}\_\text{original}\_\text{firstorder}\_10\text{Percentile }\\ -\;0.2804\;\times \text{PRE}\_\text{Original}\_\text{original}\_\text{firstorder}\_\text{Maximum} \end{array}$$

$$\begin{array}{c} CRH\;\text{Model score}\\ =0.3814\;+\;0.1261\;\times \text{ Vascular Invasion }-\;0.0255\;\times \text{ d}\_\text{tumor }\\ +\;0.0953\;\times \text{ DP}\_\text{Original}\_\text{original}\_\text{firstorder}\_10\text{Percentile }\\ -\;0.1305\;\times \text{ DP}\_\text{Original}\_\text{original}\_\text{glrlm}\_\text{ShortRunEmphasis }\\ +\;0.0726\;\times \text{ DP}\_\text{Original}\_\text{original}\_\text{firstorder}\_90\text{Percentile }\\ +\;0.1217\;\times \text{ DP}\_\text{Original}\_\text{original}\_\text{firstorder}\_\text{Maximum }\\ +\;0.0904\;\times \text{ LAP}\_\text{Original}\_\text{original}\_\text{firstorder}\_10\text{Percentile }\\ -\;0.0658\;\times \text{ PRE}\_\text{Original}\_\text{original}\_\text{firstorder}\_\text{Maximum }\\ -\;0.1812\;\times \text{ DP}\_\text{Habitat}\_3\_\text{original}\_\text{firstorder}\_\text{Kurtosis }\\ +\;0.1756 \end{array}$$

$$\begin{array}{c} \times \text{ PVP}\_\text{Habitat}\_3\_\text{original}\_\text{firstorder}\_\text{RootMeanSquared}\\ +\;0.1000\;\times \text{ PVP}\_\text{Habitat}\_1\_\text{original}\_\text{firstorder}\_\text{Minimum }\\ -\;0.0866\;\times \text{ PRE}\_\text{Habitat}\_1\_\text{original}\_\text{firstorder}\_\text{Range }\\ -\;0.0824\times \text{ PRE}\_\text{Habitat}\_2\_\text{original}\_\text{firstorder}\_\text{Skewness }\\ +\;0.0523\;\times \text{ DP}\_\text{Habitat}\_2\_\text{original}\_\text{firstorder}\_\text{Minimum} \end{array}$$

**Figure 4:**
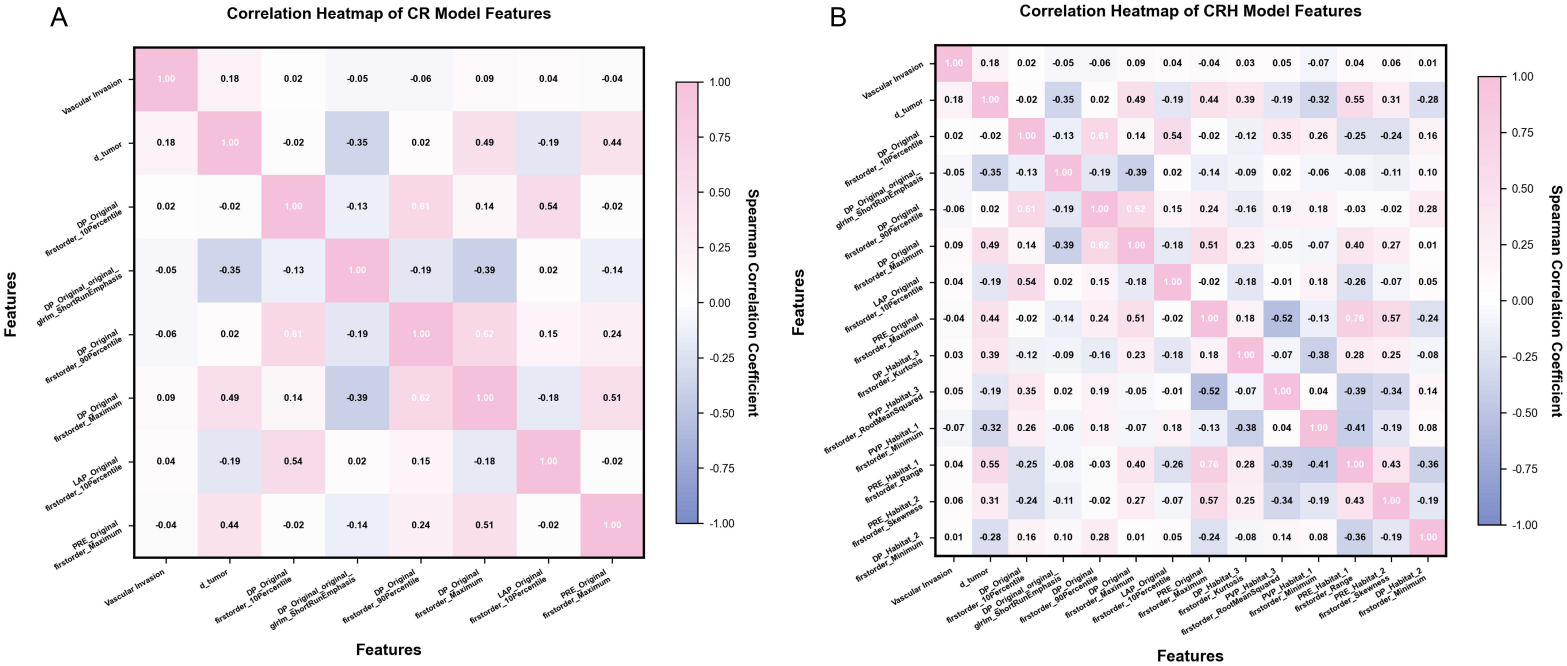
Heatmaps showing feature correlations in the two combined models. **(A)** CR model. **(B)** CRH model.

No significant redundancy or multicollinearity was observed (CR: 8 features, mean VIF = 2.445, max=4.930; CRH: 14 features, mean VIF = 2.802, max=5.990), supporting feature independence and model stability (**Figure 4**).

### 3.4 Model evaluation

**Figure 5:**
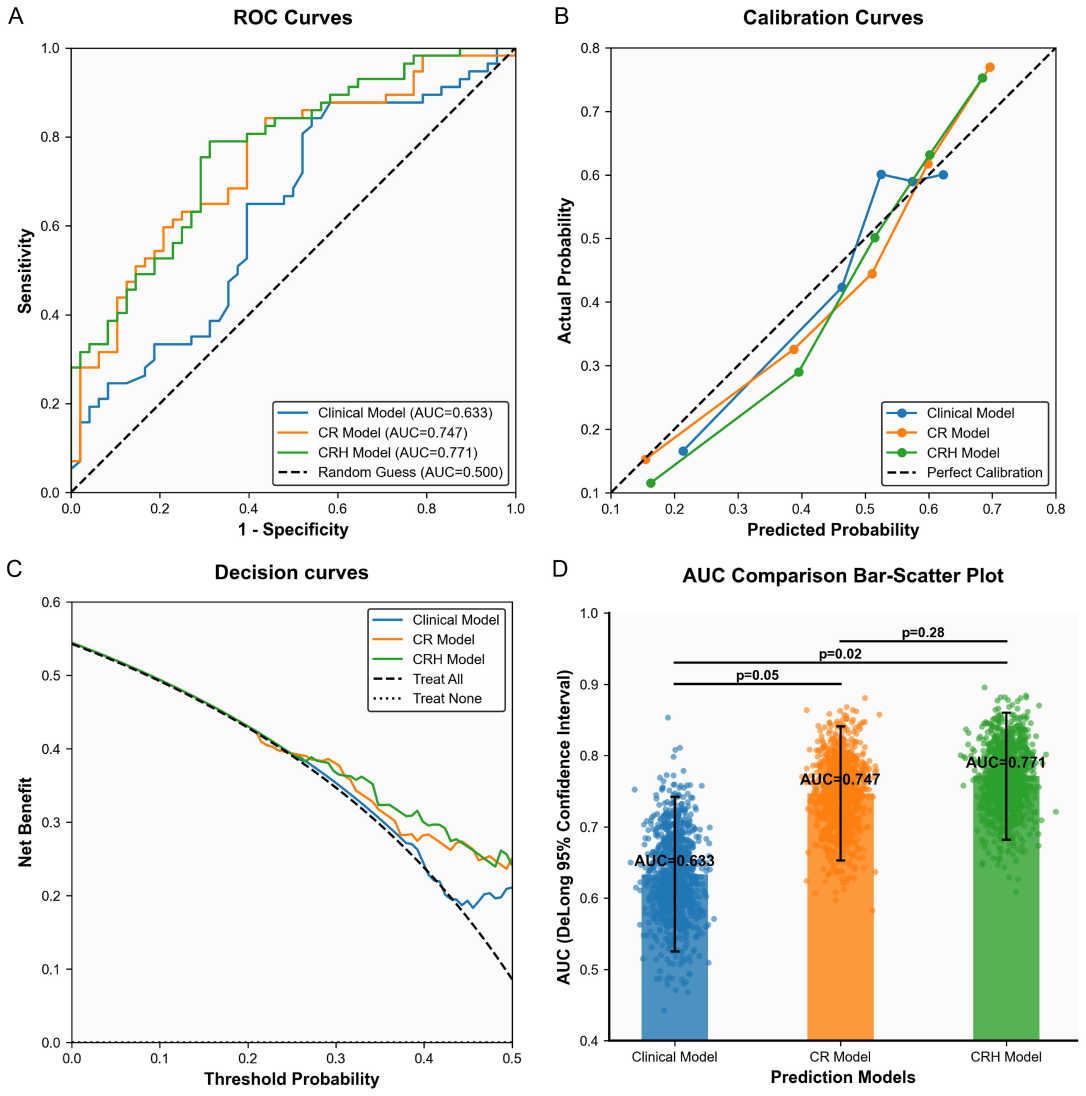
Performance evaluation of the three models. **(A)** ROC curves of each model. **(B)** Calibration curves. **(C)** Decision curves. **(D)** AUC Comparison Bar-Scatter Plot.

ROC analysis showed that the clinical, CR, and CRH models achieved AUCs of 0.633 (95% CI: 0.525–0.742), 0.747 (95% CI: 0.653–0.841), and 0.771 (95% CI: 0.682–0.860), respectively. DeLong tests confirmed that the CR model showed significantly better performance than the clinical model (Z=–1.972, p=0.05), and the CRH model performed even better (Z=–2.386, p=0.02). Although the difference between the CR and CRH models did not reach statistical significance (Z = –1.071, p = 0.28), bootstrap validation indicated stable model performance (SD of AUC: CR = 0.048, CRH = 0.046). By incorporating six additional habitat features, the CRH model enhanced the representation of tumor microenvironment heterogeneity, increasing feature dimensionality and providing more biologically meaningful prognostic information (**Figure 5**).

**Figure 6:**
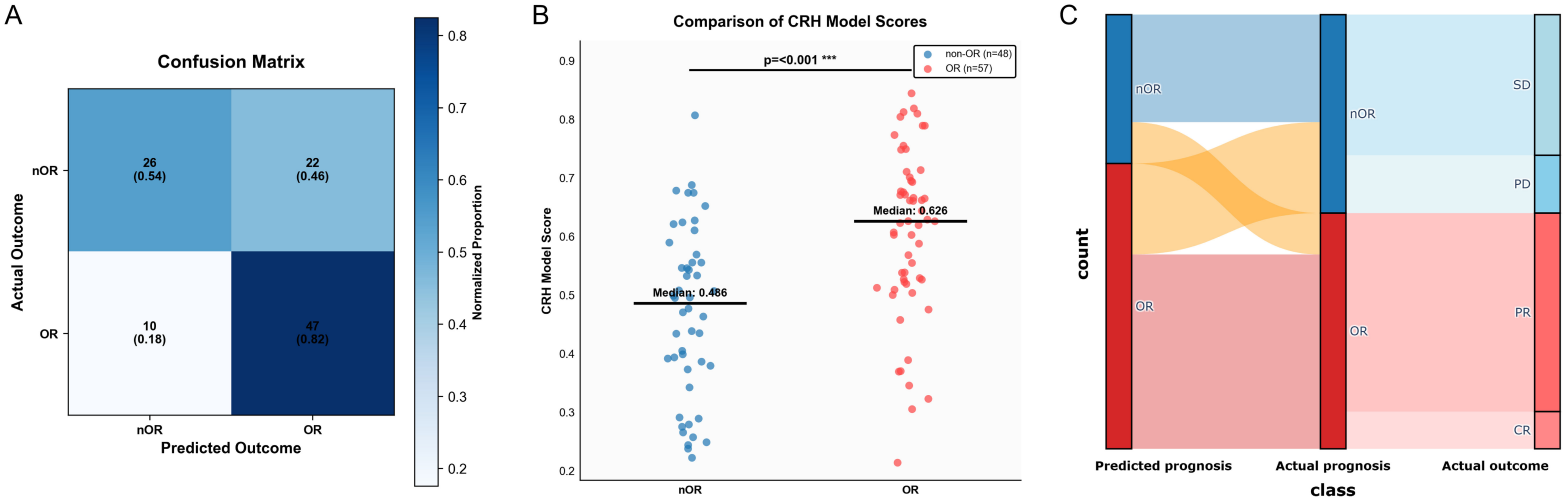
Performance evaluation of the CRH model for predicting the therapeutic response of HAIC. **(A)** Confusion matrix. **(B)** Comparison of model scores between OR and nOR groups. **(C)** Sankey diagram showing correspondence between predicted and actual treatment responses.

We validated the performance of the CRH model in classifying patients. **Figure 6A** depicts a confusion matrix with satisfactory overall accuracy. The difference in predicted risk scores between OR and nOR groups is statistically significant (p<0.001), with a median risk score of 0.626 in the OR group compared with that of 0.486 in the nOR group (**Figure 6B**). This indicated that our model can distinguish between patients with different clinical outcomes. **Figure 6C** shows the Sankey diagram analysis, which revealed good agreement between our model’s prediction of early response and patients’ actual outcomes.

## 4 Discussion

HCC is known for significant intratumoral heterogeneity and a complex tumor microenvironment, which contribute to the marked interpatient variability in response to HAIC (16, 17). Therefore, preoperative individualized treatment planning and prediction of response are crucial. To date, existing prognostic models have included laboratory-based markers (eg, AFP, DCP, and VEGF) (18–22), imaging parameters (e.g., ADC, intratumoral fat content) (23–25), and genomic features (26, 27). However, these models have been imprecise and limited in their predictive ability. Traditional radiomics-based models have shown superior performance over prior approaches (7, 28), but such analyses generally assess the entire tumor and fail to capture spatial heterogeneity within lesions. Our model includes habitat features combined with traditional radiomics features; as a result, AUC increased without impairing the stability of the model. The enriched feature set provides a more flexible and biologically informed basis for individualized prediction, supporting pre-treatment clinical decision-making.

Prior radiomics studies have frequently investigated wavelet and Gaussian filtering features (7, 29). These filtered features indicate latent image information extracted after being transformed by the original image using certain type of transformation. In this study, we only used the original image characteristics. Given our small sample size, including filtered features would have greatly increased feature dimensionality and increased overfitting risk while reducing generalizability. Furthermore, these high-dimensional abstract features often lack clear biological or clinical interpretability, and substantial redundancy exists across different filters and habitats (30–32). Compared with filtered features, habitat features derived from original images can effectively capture intratumoral heterogeneity. Meanwhile, the CT-based habitat model developed by Wu et al. (10) used arterial phase and venous phase enhancement map (enhancement maps are generated by subtracting the non-contrast image from a contrast-enhanced arterial/venous phase image). This model emphasized intratumoral perfusion characteristics and yielded biologically interpretable subregions, providing valuable insights for related research. However, it may also overlook global tumor information.

Our study used multiparametric MRI for feature extraction. This is another approach suitable for establishing HAIC response prediction models. MRI provides much richer information than CT without irradiating patients with ionizing radiation and is now recognized as an international standard method of evaluating HCC prior to treatment (2, 11). The diversity of MRI sequences enables more detailed and multidimensional characterization of tumor subregions. Although no statistically significant performance differences were observed between the CRH and CR models, the CRH model exhibited a numerically higher AUC and showed a trend toward distinguishing patients with differing actual early response outcomes, which hints at its potential clinical utility. Moreover, as suggested by several previous studies (33), peritumoral regions represent a promising new target for habitat analysis in HCC. Diversified habitat segmentation and feature extraction remain important avenues for future research in HAIC response prediction.

Several of the selected habitat features were derived from first-order statistics across different contrast-enhanced MRI phases, particularly portal venous and delayed phases, which may reflect intratumoral heterogeneity in perfusion and tissue composition. From an imaging and biological perspective, these habitat subregions may correspond to areas with variable vascular supply, necrosis, or fibrosis, leading to heterogeneous drug delivery and treatment sensitivity. This is particularly relevant for HAIC, a locoregional therapy that strongly depends on arterial blood flow and microvascular integrity, where spatial heterogeneity within the tumor may directly influence therapeutic efficacy. In addition, the present study focuses on early treatment response rather than long-term survival outcomes, as the prognosis of patients undergoing HAIC is frequently influenced by subsequent treatments, including surgery and systemic therapies; therefore, early response was chosen as the primary study endpoint to reduce potential confounding effects from post-HAIC interventions.

Our study has a few limitations worth noting. First, it’s based on retrospective data and includes only a modest number of patients, which may influence feature selection and model stability and necessitate a large-scale multi-center prospective validation. Second, only multi-phase contrast-enhanced T1WI images are used in our study. Adding other sequences, such as T2WI, may further improve model performance. Third, because the imaging data were acquired with a variety of scanners and protocols, although we have conducted preprocessing to mitigate inter-scanner variability, more standardized image acquisition and processing protocols would be helpful.

In conclusion, we developed an MRI-based Clinical–Radiomics–Habitat model for non-invasive pre-treatment prediction of early response in HCC patients undergoing HAIC treatment, which may assist clinicians in optimizing personalized therapeutic strategies.

## Data Availability

The original contributions presented in the study are included in the article/**Supplementary Material**. Further inquiries can be directed to the corresponding authors.
