# Supplementary material for: MRI-based clinical-radiomics-habitat model for predicting prognosis of hepatocellular carcinoma patients treated with HAIC
Source: Front Oncol. 2026 Jan 28;16:1764150. doi: 10.3389/fonc.2026.1764150 (PMC12890658; doi:10.3389/fonc.2026.1764150)
Supplement: Supplementary file 1 [file DataSheet1.pdf]

Supplementary Material

1 MRI Scanning Protocol

All patients underwent abdominal dynamic contrast-enhanced MRI using a 3.0 T whole-body MR scanner. Three different scanner platforms were used across institutions:

(1) SIGNA Architect AIR (GE Medical Systems, USA) with a 30AA+40PA phased-array receiver coil;

(2) uMR 790 (United Imaging Healthcare, China) with a built-in body coil (VTC); and

(3) Ingenia (Philips Medical Systems, the Netherlands) with a multi-channel phased-array coil (MULTI COIL).

For each system, a breath-hold 3D T1-weighted gradient-echo sequence was employed for dynamic imaging:

On the GE scanner, a LAVA sequence was used with the following parameters: TR = 3.788 ms, TE = 1.572 ms, slice thickness = 3.93 mm, slice spacing = 2 mm, matrix = 512 × 512, pixel spacing = 0.7813 × 0.7813 mm, flip angle = 120°, inversion time = 24 ms, bandwidth = 434.023 Hz/pixel.

On the United Imaging scanner, a t1_quick3d sequence was applied with: TR = 3.45 ms, TE = 1.59 ms, slice thickness = 3 mm, slice spacing = 3 mm, matrix = 348 × 480, pixel spacing = 0.833 × 0.833 mm, flip angle = 10°, pixel bandwidth = 650 Hz/pixel, acquisition duration = 15.23 s.

On the Philips scanner, an mDIXON-W sequence was performed with: TR = 4.405 ms, TE = 1.38 ms, slice thickness = 3.5 mm, slice spacing = 1.75 mm, matrix = 528 × 528, pixel spacing = 0.76 × 0.76 mm, flip angle = 10°, acquisition duration = 13.6 s.

In all cases, contrast-enhanced images were acquired after intravenous injection of gadopentetate dimeglumine (Magnevist®, Bayer AG, Germany) at a standard dose of 0.1 mmol/kg body weight, covering the arterial, portal venous, and delayed phases. For radiomics analysis, the pre-contrast (mask) T1-weighted image along with the three post-contrast phases (arterial, venous, and delayed) from each patient were selected, resulting in four multiphase MRI sequences per subject.

2 Radiomics and Habitat Features

For each patient, a total of 428 radiomics features were extracted from the four MRI sequences, with 107 radiomics features derived from each sequence. The feature names are listed below:

Original_original_firstorder_10Percentile

Original_original_firstorder_90Percentile
Original_original_firstorder_Energy
Original_original_firstorder_Entropy
Original_original_firstorder_InterquartileRange
Original_original_firstorder_Kurtosis
Original_original_firstorder_Maximum
Original_original_firstorder_Mean
Original_original_firstorder_MeanAbsoluteDeviation
Original_original_firstorder_Median
Original_original_firstorder_Minimum
Original_original_firstorder_Range
Original_original_firstorder_RobustMeanAbsoluteDeviation
Original_original_firstorder_RootMeanSquared
Original_original_firstorder_Skewness
Original_original_firstorder_TotalEnergy
Original_original_firstorder_Uniformity
Original_original_firstorder_Variance
Original_original_glcm_Autocorrelation
Original_original_glcm_ClusterProminence
Original_original_glcm_ClusterShade
Original_original_glcm_ClusterTendency
Original_original_glcm_Contrast
Original_original_glcm_Correlation
Original_original_glcm_DifferenceAverage
Original_original_glcm_DifferenceEntropy
Original_original_glcm_DifferenceVariance
Original_original_glcm_Id
Original_original_glcm_Idm
Original_original_glcm_Idmn
Original_original_glcm_Idn
Original_original_glcm_Imc1
Original_original_glcm_Imc2
Original_original_glcm_InverseVariance
Original_original_glcm_JointAverage
Original_original_glcm_JointEnergy
Original_original_glcm_JointEntropy
Original_original_glcm_MaximumProbability
Original_original_glcm_MCC
Original_original_glcm_SumAverage
Original_original_glcm_SumEntropy
Original_original_glcm_SumSquares
Original_original_gldm_DependenceEntropy
Original_original_gldm_DependenceNonUniformity
Original_original_gldm_DependenceNonUniformityNormalized

Original_original_gldm_DependenceVariance
Original_original_gldm_GrayLevelNonUniformity
Original_original_gldm_GrayLevelVariance
Original_original_gldm_HighGrayLevelEmphasis
Original_original_gldm_LargeDependenceEmphasis
Original_original_gldm_LargeDependenceHighGrayLevelEmphasis
Original_original_gldm_LargeDependenceLowGrayLevelEmphasis
Original_original_gldm_LowGrayLevelEmphasis
Original_original_gldm_SmallDependenceEmphasis
Original_original_gldm_SmallDependenceHighGrayLevelEmphasis
Original_original_gldm_SmallDependenceLowGrayLevelEmphasis
Original_original_glrlm_GrayLevelNonUniformity
Original_original_glrlm_GrayLevelNonUniformityNormalized
Original_original_glrlm_GrayLevelVariance
Original_original_glrlm_HighGrayLevelRunEmphasis
Original_original_glrlm_LongRunEmphasis
Original_original_glrlm_LongRunHighGrayLevelEmphasis
Original_original_glrlm_LongRunLowGrayLevelEmphasis
Original_original_glrlm_LowGrayLevelRunEmphasis
Original_original_glrlm_RunEntropy
Original_original_glrlm_RunLengthNonUniformity
Original_original_glrlm_RunLengthNonUniformityNormalized
Original_original_glrlm_RunPercentage
Original_original_glrlm_RunVariance
Original_original_glrlm_ShortRunEmphasis
Original_original_glrlm_ShortRunHighGrayLevelEmphasis
Original_original_glrlm_ShortRunLowGrayLevelEmphasis
Original_original_glszm_GrayLevelNonUniformity
Original_original_glszm_GrayLevelNonUniformityNormalized
Original_original_glszm_GrayLevelVariance
Original_original_glszm_HighGrayLevelZoneEmphasis
Original_original_glszm_LargeAreaEmphasis
Original_original_glszm_LargeAreaHighGrayLevelEmphasis
Original_original_glszm_LargeAreaLowGrayLevelEmphasis
Original_original_glszm_LowGrayLevelZoneEmphasis
Original_original_glszm_SizeZoneNonUniformity
Original_original_glszm_SizeZoneNonUniformityNormalized
Original_original_glszm_SmallAreaEmphasis
Original_original_glszm_SmallAreaHighGrayLevelEmphasis
Original_original_glszm_SmallAreaLowGrayLevelEmphasis
Original_original_glszm_ZoneEntropy
Original_original_glszm_ZonePercentage
Original_original_glszm_ZoneVariance
Original_original_ngtdm_Busyness

Original_original_ngtdm_Coarseness
Original_original_ngtdm_Complexity
Original_original_ngtdm_Contrast
Original_original_ngtdm_Strength
Original_original_shape_Elongation
Original_original_shape_Flatness
Original_original_shape_LeastAxisLength
Original_original_shape_MajorAxisLength
Original_original_shape_Maximum2DDiameterColumn
Original_original_shape_Maximum2DDiameterRow
Original_original_shape_Maximum2DDiameterSlice
Original_original_shape_Maximum3DDiameter
Original_original_shape_MeshVolume
Original_original_shape_MinorAxisLength
Original_original_shape_Sphericity
Original_original_shape_SurfaceArea
Original_original_shape_SurfaceVolumeRatio
Original_original_shape_VoxelVolume

Additionally, a total of 1,140 habitat features were extracted per patient across the four sequences. For each sequence, features were obtained from three HABITAT subregions. Within each HABITat, 93 radiomics features (excluding shape features) were extracted, along with the absolute volume of the habitat and its percentage relative to the whole-tumor volume, resulting in 95 features per habitat per sequence. The feature names are listed below:

Original_firstorder_10Percentile
Original_firstorder_90Percentile
Original_firstorder_Energy
Original_firstorder_Entropy
Original_firstorder_InterquartileRange
Original_firstorder_Kurtosis
Original_firstorder_Maximum
Original_firstorder_MeanAbsoluteDeviation
Original_firstorder_Mean
Original_firstorder_Median
Original_firstorder_Minimum
Original_firstorder_Range
Original_firstorder_RobustMeanAbsoluteDeviation
Original_firstorder_RootMeanSquared
Original_firstorder_Skewness
Original_firstorder_TotalEnergy
Original_firstorder_Uniformity
Original_firstorder_Variance
Original_glcm_Autocorrelation

Original_glcm_ClusterProminence
Original_glcm_ClusterShade
Original_glcm_ClusterTendency
Original_glcm_Contrast
Original_glcm_Correlation
Original_glcm_DifferenceAverage
Original_glcm_DifferenceEntropy
Original_glcm_DifferenceVariance
Original_glcm_Id
Original_glcm_Idm
Original_glcm_Idmn
Original_glcm_Idn
Original_glcm_Imc1
Original_glcm_Imc2
Original_glcm_InverseVariance
Original_glcm_JointAverage
Original_glcm_JointEnergy
Original_glcm_JointEntropy
Original_glcm_MCC
Original_glcm_MaximumProbability
Original_glcm_SumAverage
Original_glcm_SumEntropy
Original_glcm_SumSquares
Original_glrlm_GrayLevelNonUniformity
Original_glrlm_GrayLevelNonUniformityNormalized
Original_glrlm_GrayLevelVariance
Original_glrlm_HighGrayLevelRunEmphasis
Original_glrlm_LongRunEmphasis
Original_glrlm_LongRunHighGrayLevelEmphasis
Original_glrlm_LongRunLowGrayLevelEmphasis
Original_glrlm_LowGrayLevelRunEmphasis
Original_glrlm_RunEntropy
Original_glrlm_RunLengthNonUniformity
Original_glrlm_RunLengthNonUniformityNormalized
Original_glrlm_RunPercentage
Original_glrlm_RunVariance
Original_glrlm_ShortRunEmphasis
Original_glrlm_ShortRunHighGrayLevelEmphasis
Original_glrlm_ShortRunLowGrayLevelEmphasis
Original_glszm_GrayLevelNonUniformity
Original_glszm_GrayLevelNonUniformityNormalized
Original_glszm_GrayLevelVariance
Original_glszm_HighGrayLevelZoneEmphasis
Original_glszm_LargeAreaEmphasis

Original_glszm_LargeAreaHighGrayLevelEmphasis
Original_glszm_LargeAreaLowGrayLevelEmphasis
Original_glszm_LowGrayLevelZoneEmphasis
Original_glszm_SizeZoneNonUniformity
Original_glszm_SizeZoneNonUniformityNormalized
Original_glszm_SmallAreaEmphasis
Original_glszm_SmallAreaHighGrayLevelEmphasis
Original_glszm_SmallAreaLowGrayLevelEmphasis
Original_glszm_ZoneEntropy
Original_glszm_ZonePercentage
Original_glszm_ZoneVariance
Original_gldm_DependenceEntropy
Original_gldm_DependenceNonUniformity
Original_gldm_DependenceNonUniformityNormalized
Original_gldm_DependenceVariance
Original_gldm_GrayLevelNonUniformity
Original_gldm_GrayLevelVariance
Original_gldm_HighGrayLevelEmphasis
Original_gldm_LargeDependenceEmphasis
Original_gldm_LargeDependenceHighGrayLevelEmphasis
Original_gldm_LargeDependenceLowGrayLevelEmphasis
Original_gldm_LowGrayLevelEmphasis
Original_gldm_SmallDependenceEmphasis
Original_gldm_SmallDependenceHighGrayLevelEmphasis
Original_gldm_SmallDependenceLowGrayLevelEmphasis
Original_ngtdm_Busyness
Original_ngtdm_Coarseness
Original_ngtdm_Complexity
Original_ngtdm_Contrast
Original_ngtdm_Strength
Volume
Habitat_Percent
